# Readability of written medicine information materials in Arabic language: expert and consumer evaluation

**DOI:** 10.1186/s12913-018-2944-x

**Published:** 2018-02-27

**Authors:** Sinaa Al Aqeel, Norah Abanmy, Abeer Aldayel, Hend Al-Khalifa, Maha Al-Yahya, Mona Diab

**Affiliations:** 1 Clinical Pharmacy Department, College of Pharmacy, King Saud University, Riyadh, Kingdom of Saudi Arabia; 2 Information Technology Department, College of Computer and Information Sciences, King Saud University, Riyadh, Kingdom of Saudi Arabia; 3 Department of Computer Science, SEAS, The George Washington University, Washington, USA

**Keywords:** Patient education, Consumer Health Information, Readability, Annotation, Arabic Language

## Abstract

**Background:**

Written Medicine Information (WMI) is one of the sources that patients use to obtain information concerning medicine. This paper aims to assess the readability of two types of WMIs in Arabic language based on vocabulary use and sentence structure using a panel of experts and consumers.

**Methods:**

This is a descriptive study. Two different types of materials, including the online text from King Abdullah Bin Abdulaziz Arabic Health Encyclopaedia (KAAHE) and medication leaflets submitted by the manufacturers to the Saudi Food and Drug Authority (SFDA) were evaluated. We selected a group of sentences from each WMI. The readability was assessed by experts (*n* = 5) and consumers (*n* = 5). The sentence readability of each measured using a specific criteria and rated as 1 = easy, 2 = intermediate, or 3 = difficult.

**Results:**

A total of 4476 sentences (SFDA 2231; KAHEE 2245) extracted from websites or patient information leaflets on 50 medications and evaluated. The majority of the vocabulary and sentence structure was considered easy by both expert (SFDA: 68%; KAAHE: 76%) and consumer (SFDA: 76%; KAAHE: 84%) groups. The sentences with difficult or intermediate vocabulary and sentence structure are derived primarily from the precautions and side effects sections.

**Conclusions:**

The SFDA and KAAHE WMIs are easy to read and understand as judged by our study sample. However; there is room for improvement, especially in sections related to the side effects and precautions.

## Background

Written Medicine Information (WMI) is one of the sources that patients use to obtain information concerning medicine. Evidence suggests that patients use the WMI to know more about their medication, to decide whether to take the medicine, to reassure themselves and to comply with therapy [1]. Various regulatory efforts have been suggested in the United State, Europe and Australia to improve the WMI’s presentation and usability [2]. Despite those efforts, the evaluation of WMIs reveals various problems, such as texts that are complex and difficult to understand, low readability, the use of small font size, lengthy sentences, and few illustrations [3–5].

Garner et al. [6] proposed a framework for the evaluation of written patient information, which involves three discrete but interrelated process, such as readability, comprehensibility, and communicative effectiveness. Readability predicts the relative ease with which a reader can assign meanings to words and phrases. Readability has both a visual and linguistic aspect. The visual aspects of readability involve font size, use of highlights, colours, and graphics. The linguistic aspects of readability include the length and syllabic make-up of words, as well as their familiarity to readers with a specified level of education. The comprehensibility is defined as the readers’ capacity to assign contextually relevant meanings to the words. The communicative effectiveness is a function of the readers’ cognitions (e.g., expectations, understandings), affect (e.g., relief, concern, worry), intentions and behaviour (e.g., taking a pill before eating) [6].

When assessing the readability of WMIs, the majority of related research utilises readability formulae, such as the Simple Measure of Gobbledygook (SMOG) and the Flesch-Kincaid grade level (FKGL) [5]. However, the use of such formulae disregards sentence structure, overall text organisation, and the consumer’s prior knowledge and understanding of the text [5]. Furthermore, polysyllabic words, such as medical terms and drug names, may artificially amplify the literacy skills required to comprehend the material [5, 6].

To complement the use of readability formulas, a number of approaches were developed for English language written materials. For instance, a readability scoring algorithm, which attempts to take aspects of text features (i.e., number of characters/word), syntactic aspects (i.e., parts of speech [POS], such as noun, verb, adverb, and adjective extracted), semantic features (i.e., average term and concept familiarity scores), and cohesion (i.e., number of overlapping concepts in adjacent sentences), was developed and applied to test the readability of medical texts, such as clinical trials information [7], medical documents [8], and patient information leaflets [9]. Others have developed a classifier, which is a machine learning technique that uses an algorithm to distinguish between three difficulty levels in documents based exclusively on the vocabulary used and was applied for health information websites on melanoma, depression, and prostate cancer [10].

Little is known about the readability of Arabic WMIs. The limited published research that is available examined understanding [11, 12], adequacy of content and format [13], and accuracy of content [14]. To our knowledge, only two studies examined readability of package inserts using Flesch-Kincaid grade level formula [15, 16]. In addition to the previously discussed limitation of using reading formulas to assess readability of WMI, the commonly used readability formulas d not produce accurate results in certain languages such as Arabic [17]. This paper attempts to describe an approach to measure Arabic WMI readability other than formulas. This study aimed to assess the readability of two types of WMIs in Arabic language based on vocabulary use and sentence structure using a panel of expert and consumers. The study is part of a large project for developing an automated Arabic WMI tagging tool, which leverages artificial intelligence algorithms to predict the readability level of new WMI materials automatically. To construct a readability prediction system, three steps were followed. First, a readability corpus containing health text material was composed. Second, readability assessments for this corpus were acquired. Finally, based on the acquired readability assessments, prediction tasks were performed. The first and final steps are described elsewhere [18, 19]. The second step, i.e., determining the readability, is discussed in this paper.

## Methods

This is a descriptive study conducted in Riyadh, Saudi Arabia. The following sections will describe methods of selection WMI materials, building the annotation corpus, participants’ characteristics, annotation procedure, readability measurement and data analysis.

### Selection of WMI materials

We selected two types of WMI materials. An online WMI obtained from the King Abdullah Bin Abdulaziz Arabic Health Encyclopaedia (KAAHE) [20] and patient information leaflets prepared by the manufactures and submitted to the Saudi Food and Drug Authority (SFDA) [21]. KAAHE is a governmental online Arabic public health encyclopaedia. The content of the KAAHE materials was developed in collaboration with international agencies, such as the UK National Health services and Patient Education Institute. The SFDA is an independent governmental body corporation. Pharmaceutical companies must submit a Summary of Product Characteristics (SmPC), labelling information and, as of August 2011, information leaflets (PIL) to the SFDA during the drug registration process. We asked both agencies, SDFA and KAAHE, for medication information materials without specifying the name of the medications nor the prescription status of the medications. The first batch of WMI we received were used as the sample for this project. We implemented no exclusion criteria to received WMI. SDFA and KAAHE provided us with soft copies (either Word or Adobe pdf) of the Arabic version of the WMI materials and permission to utilise them in the study.

For the first stage of our project, we assessed the readability of 94 WMI materials (SFDA: 47, KAAHE: 47) corresponding to 50 medications. Forty-four medications were similar between both sources of SDFA and KAAHE data, while six were unmatched medications (SFDA (*n* = 3); KAAHE (*n* = 3)). The material collected covers both the prescription-only medications and over-the-counter medications with different therapeutic indications and pharmaceutical forms.

### Building the annotation corpus

Readability assessment could be performed at the document level, where the whole document readability is assessed, or at the sentences level. In this study, we opted for a sentence-level assessment.

To create a balanced and manageable set, we selected a group of sentences, rather than including all of the sentences in the document. The WMI from SFDA and KAAHE have a different layout and use different subheadings. To ensure the quality and consistency of the extraction process, a list of subheadings and number of sentences to be extracted from each subheading was created. Under each subheading, the first, second and/or third sentences, which were defined as a group of words that ends with a period, were extracted, regardless of whether they were written as bullets or in paragraph form. The number of sentences extracted from each section was different. Three sentences were extracted from lengthy sections (indications, contraindications, precautions and side effects), two sentences from intermediate length sections (drug-food interaction, drug-drug interaction, and storage instructions), and one sentence from the rest of the sections. The sentences were extracted by a computer science student and entered manually (copy and paste) into a form created using Google forms. Sentences extracted from one WMI were grouped together in one evaluation form. A random selection of extracted sentences was reviewed by two of the pharmacists (SA, NA).

### Participants

The readability was assessed by two cohorts: experts (*n* = 5) and consumers (*n* = 5). The number of annotators was selected based on previous similar research which involved 2 to 6 annotators [10, 22]. The expert participants were comprised of physicians (*n* = 1), pharmacists (*n* = 2), and health education specialists (*n* = 2). The consumers were individuals with no health educational background and with a university-level education. The participants were recruited using convenience sampling. All participants were contacted by one of the authors (NA) who explained the purpose of the study and the method of annotation and instructions.

### Annotation procedure

Participants were sent an email with a link to the study website. The email explained that participation is voluntary and participants could withdraw at any time. The email also stressed confidentiality and anonymous data analysis procedures. Clicking on the study website link indicated the participant willingness to take part in the study. The website included an “introductory” page containing the method of annotation and instructions. Next, the participants proceeded to the annotation page containing the extracted sentences.

Each WMI was evaluated by one expert and one consumer annotator. Ten WMIs were evaluated by four evaluators (two experts and two consumers) to enable calculation of the inter-annotator agreement (IAA) measurement. One of the authors (SA) and one consumer acted as adjudicators in cases of disagreement between the annotators.

### Readability measurement

Each sentence was rated as (1 = easy; 2 = intermediate; or 3 = difficult) from a drop-down menu. Table 1 provides an overview of how “easy,” “intermediate,” and “difficult” are defined based on vocabulary use and sentence structure. These definitions are adapted from the definitions of the readability difficulty levels of online health information for both experts and consumers, as defined by Leory et al. [10]. We used the definitions after modifying them to be used at the sentence level, rather than the document level. The modified definitions were translated to Arabic by one author (SA) and checked for accurate translation by the other authors. The definitions were piloted on two documents by the authors.

**Table 1:** Readability level definitions^a^

	Expert	Consumer
Easy	The sentence contains few medical vocabulary and all used by the average consumer. The syntactic constructions of the sentence is similar to that used by the average consumer. The average consumer can understand the sentence without any help.	The sentence contains few medical vocabulary and all familiar to you. The syntactic constructions of the sentence is similar to structure that you would write. You can understand the sentence without any help.
Intermediate	The sentence contains medical vocabulary used in consumer health education. The syntactic constructions of the sentence is used typically in consumer health education materials. The sentence is understood as consumer health education.	The sentence contains medical vocabulary that some are unfamiliar to you. The sentence has a structure that you can understand. You can understand the sentence with the help of references or your friends or family.
Difficult	The sentence contains medical vocabulary typically used by health professionals. The syntactic constructions of the sentence is typically used by health professionals. Only medical professionals can understand this sentence.	The sentence contains many medical terms you do not understand. The sentence has a structure that health professionals would write. Only medical professionals can understand this sentence.

^a^Adapted from reference [10]

### Data analysis

All data was entered into a Microsoft Excel (version 2013, Microsoft, Redmond, WA) spreadsheet for data entry, data retrieval, and analysis. Descriptive analyses were calculated for all the quantitative values. IAA among the annotators was calculated using the Kappa statistic or Fleiss Kappa as appropriate. The t-test was used to examine the difference between SFDA and KAHEE readability score means. A *p*-value of < 0.05 was considered statistically significant. We calculated the average word length (AWL) and average sentence length (ASL) using the LEN and SUBSTITUTE functions in Excel. AWL is defined as the number of characters per word (number of characters divided by the number of words), where ASL is defined as the average number of words in a sentence (number of words divided by the number of sentences).

## Results

A total of 4476 sentences were evaluated by expert and consumers (SFDA 2231; KAAHE 2245) from 94 WMIs. The average IAA (calculated using Cohen’s Kappa) was 0.243 (min 0.006, max 0.493) within the annotators from the same group. The interrater agreement between the expert and consumers annotators (calculated using Fleiss Kappa) was 0.20 (min 0.15, max 0.323).

The mean scores for SFDA and KAAHE WIMs assed by experts were 1.39 (SD 0.26) and 1.31 (SD 0.18), respectively. The mean scores for SFDA and KAHEE WIMs, as assessed by consumers, were 1.32 (SD 0.23) and 1.21 (SD 0.14), respectively. The differences between SFDA scores and KAAHE scores were statistically significant in the consumer group (t = 2.70, df = 92, *P* = 0.001) and the expert group (t = 1.71, df = 92, *P* = 0.089). As shown in Table 2, the experts and consumers considered 68% and 76% of the SFDA sentences as easy for their vocabulary use and sentence structure, respectively. For the KAHEE, the vocabulary use and sentence structure were considered easy in 76% and 84% of the sentences by experts and consumers, respectively.

**Table 2:** Expert and consumer readability assessment for sentences (*n* = 4476), easy, intermediate (inter), and difficult (diff)

	SFDA (*n* = 2231)			KAHEE (*n* = 2245)		
	Easy n (%)	Inter. n (%)	Diff n (%)	Easy n (%)	Inter. n (%)	Diff n (%)
Expert	1518 (68)	534 (24)	179 (8)	1706 (76)	384 (17)	155 (7)
Consumer	1691 (76)	369 (17)	171 (8)	1886 (84)	247 (11)	112 (5)

As shown in Table 3, the distribution of the difficult sentences varied by sections for the WMIs. For the SFDA, the majority of the difficult-to-read sentences came from the “What the (product name) is and what it is used for” section, which describes the active ingredients, mode of action, and indications. This section was followed by the “Before you take (product name)” section, which describes the precautions and contraindications, which was followed by the “possible side effects” section. For KAHHE, the majority of the difficult-to-read sentences came from the “drug-drug interaction” section followed by the “therapeutic classification and dosage” section and the “mechanism of action” section thereafter.

**Table 3:** Distribution of difficult and intermediate sentences by WMIs sections

	Difficult		Intermediate	
WMIs Section	Expert	Consumers	Expert	Consumers
SFDA				
What the (product name) is and what it is used for	41	40	114	82
Before you take (product name)	70	69	168	145
Possible side effects	39	25	130	79
How to use	15	9	82	41
Storage	0	3	9	1
Ingredients	14	25	29	21
Total	179	171	532	369
KAHHE				
Therapeutic classification and dosage	48	21	56	41
Mechanism of action	45	17	57	39
Contraindications	3	6	38	17
drug–drug interaction	54	61	103	87
Precautions before using this medication	3	1	29	9
Common side effects	0	4	7	14
Reasons to call your doctor	0	0	34	11
Others	5	4	62	23
Total	158	115	386	241

We explored the relationship between the AWL and ASL and the difficulty level. As shown in Table 4, longer sentences tend to be rated more difficult.

**Table 4:** Proportion of easy, intermediate (inter), and difficult (diff) sentences by AWL and ASl

	SFDA (*n* = 2231)			KAHEE (*n* = 2245)			ALL (*n* = 4476)		
	Easy	Inter	Diff	Easy	Inter.	Diff	Easy	Inter.	Diff
Expert									
AWL	5.58	5.78	6.15	6.42	6.77	7.39	6.00	6.21	6.21
ASL	16.21	20.63	23.37	14.02	22.27	27.48	15.05	21.32	25.28
Non-Expert									
AWL	5.58	5.95	6.14	6.41	7.07	7.94	5.99	6.42	6.94
ASL	17.17	20.47	19.00	15.22	22.01	23.14	16.14	21.09	20.64

*AWL* Average Word Length, *ASL* Average Sentence Length

## Discussion

As part of a larger project, the aim of our study was to measure the readability of WMIs in Arabic language based on vocabulary use and sentence structure using a panel of experts and consumers. Specifically, we asked five experts and five consumers to read and annotate the difficulty level of 94 WMIs available for Saudi Arabia consumers.

Our main finding indicates that the majority of WMIs examined are easy to read as judged by our panel of experts and consumers. This finding reflects the approaches used by the KAAHE and SFDA to develop their WMIs. For instance, the new guidelines of the SFDA on the development of package inserts were adapted from the European Medical Agency (EMA) and address many of the limitations identified in research examining the old versions of the package inserts in Saudi Arabia [13]. However, there is room for improvement, as approximately 16%–32% of the examined sentences were judged by our panel as difficult or intermediate for their vocabulary use and sentence structure and cannot be understood by the consumer without help.

As shown in Tables 3 and 4, difficult and intermediate difficulty sentences are predominantly long sentences in the precautions and side effects sections. One explanation could be that these two sections contain many medical terms and technical words. Previous studies have shown that medical terminology is unfamiliar to patients and difficult to understand [23, 24]. One promising approach to overcome this problem is text simplification. There are many approaches to the simplification task, including lexical (i.e., identifying and replacing complex words with simpler substitutes), syntactical (i.e., identifying grammatical complexities in a text and rewriting these into simpler structures), statistical machine translation and hybrid techniques [25]. Published work on the simplification of medical texts is beginning to accumulate [26, 27]. Further research examining the simplification of Arabic WMIs is warranted.

Our findings suggest that the experts considered more WMIs to be too difficult than did the consumers. This unexpected finding has been observed in previous literature. In a US study by Leory et al., an expert and a consumer assessed the vocabulary, structure, and overall appearance of 90 documents from commercial Web sites, government/educational Web sites, and those provided by consumer groups themselves. The experts considered the pages more difficult for a consumer than the consumer did. For instance, the expert considered vocabulary to be difficult for the average consumer in 10% of the government/non-profit pages and 33% of the commercial pages, while the consumer considered the government/non-profit pages to have difficult vocabulary in 7% of the cases and the commercial pages in 23% of the cases [10]. One explanation could be that the experts may be underestimating the average consumer, while the consumers may be overestimating themselves. As per our instructions, experts were asked to assess the difficulty for an average WMI consumer audience, while all of our consumers have high qualification, which may also explain the results.

In Europe, it is a legal requirement for package inserts to be user-tested, as Article 59(3) of the European Council directive states that “the package leaflet shall reflect the results of consultations with target patient groups to ensure that it is legible, clear and easy to use” [28]. The user tests try to find whether participants in the consultation (potential patients) can find and understand key messages in the package inserts, which will ensure safe and effective use of the medicine. The goal is for 90% of the test participants to be able to find the information and for 90% of that group to be able to express it in their own words. We recommend that user testing should also be a requirement by the SFDA and KAAHE.

One strength in our study is involving consumers in the evaluation process. Our study only evaluated the readability of the SFDA and KAAHE written information, future research should examine the comprehensibility of this information from the consumer perspective. As we discussed in the introduction, most of the related research utilises a readability formula to assess readability. Our study is one of several that utilised a human annotator to assess readability. Previous studies that used humans to evaluate readability utilised different scales, such as a scale of 1, meaning “understood by anyone with basic literacy,” to 7, meaning “understood only by someone with professional education” [22], a scale of 1 (very easy) to 4 (very difficult) [29], or a scale of easy, intermediate, and difficult based on the vocabulary, structure, and overall appearance [10]. In our study, we adapted the last scale because we think it is comprehensive, as it examines the vocabulary difficulty, as well as the sentence structure. Another strength in our study is that we examined two type of WMIs: patient information leaflets and websites. To the best of our knowledge, KAAHE is the only governmental, non-commercial evidence-based health information website available in the Arabic language, which is one of the most spoken language in the world. Future studies should also examine the readability of other types of WMIs, such as discharge reports, diagnostic procedure instructions, and commercial websites.

The current study has several limitations. First, we assessed the readability on the sentence level rather than the document level. Although the extracted sentences used in our corpus are meaningful by themselves, we acknowledge that this approach ignores the cohesion or coherence between sentences. Cohesion is an important factor in readability [22]. Additionally, this approach ignores the document’s style (such as font size), which also affects the document’s readability. Second, we did not validate the test-retest reliability of the evaluation tool to ensure that the level of difficulty assigned by an annotator was relatively stable across the two time periods. Third, the calculated inter-annotator agreement is relatively low. One explanation could be the different background and experience of our sample. Fourth, the high education level of our consumer annotators limit the generalisability of the results to general consumers.

## Conclusions

Our results suggest that the Arabic language SFDA and KAAHE WMIs are easy to understand as judged by our study sample. However, there is room for improvement, especially in sections related to side effects and precautions.

## Abbreviations

ASL: Average Sentence Length
AWL: Average Word Length
KAAHE: King Abdullah Bin Abdulaziz Arabic Health Encyclopaedia
SFDA: Saudi Food and Drug Authority
WMI: Written Medicine Information
